# Aerosol mixing state matters for particles deposition in human respiratory system

**DOI:** 10.1038/s41598-018-27156-z

**Published:** 2018-06-11

**Authors:** Joseph Ching, Mizuo Kajino

**Affiliations:** 1 Meteorological Research Institute, Japan Meteorological Agency, 1-1 Nagamine, Tsukuba, Ibaraki, 305-0052 Japan; 2 Faculty of Life and Environmental Sciences, University of Tsukuba, 1-1-1 Tennodai, Tsukuba, Ibaraki, 305-8577 Japan

## Abstract

Aerosol particles emitted from various human activities deteriorate air quality and are suggested to increase public health risk. Numerous studies have emphasized the relationship between the mass and/or number concentration of aerosols (or commonly known as particulate matter (PM)) in the atmosphere and the incidence of respiratory and cardiovascular diseases, while very few have examined the deposition efficiency of inhaled particles in the respiratory tract. We present the first examination of particles deposition based on, detailed simulation of aerosol physico-chemical properties by a recently developed particle-resolved aerosol model and the mixing state dependent hygrosocpic growth and deposition computed at particle-level by deposition model. Furthermore, we elucidate the impact of mixing state on deposition efficiency by using a recently introduced aerosol mixing state index. We find that without considering mixing-state-dependent hygroscopic growth of particles leads to overestimation of deposition efficiency; whereas considering an average mixing state leads to underestimation of 5% to 20% of soot particle deposition efficiency in human alveoli. We conclude that aerosol mixing state, which evolves during the interaction between atmospheric chemistry and meteorology, is important for the comprehensive evaluation of air quality and its implication to public health requires further investigation.

## Introduction

Aerosol particles emitted from both natural and anthropogenic sources are widespread throughout the world. They influence the Earth’s energy budget and therefore climate system by directly interacting with solar and terrestrial radiation or indirectly modifying clouds^1–3^. Commonly known as particulate matter and abbreviated as PM, aerosols are blamed for deteriorating visibility and air quality. When facilitated by certain meteorological conditions and/or city landscapes, the number concentration of aerosol, whether from natural or anthropogenic sources, can reach an elevated level. Air pollution presents a human health risk^4^ and is associated with mortality^5,6^. Particulate matter with a diameter less than 2.5 micrometers, or PM_2.5_, is able to penetrate deeply into the cardiovascular system and lung, hence presenting a tremendous human health risk^7–9^. According to recent air quality model results released by the World Health Organization (WHO) in September 2016^10^, approximately three millions deaths in 2012 worldwide were related to outdoor air pollution. The risk of cardiovascular disease, stroke, lung cancer, and chronic and acute respiratory disease is associated with air pollution^10^. WHO’s report on reducing global health risks suggested that reducing emissions of some short-lived climate pollutants, such as black carbon (BC), could benefit human health by reducing air-pollution-related disease and death^11,12^. Furthermore, the International Agency for Research on Cancer (IARC) of WHO in its 2013 assessment noted the carcinogenic nature of outdoor air pollution to which humans are exposed^13^.

Numerous studies have indicated that the negative impacts of aerosol particles on human health could be severe, leading to respiratory diseases and cancer in particular^5,7,9,14^. Most previous studies have focused on the impact of the number concentration, mass concentration, size and composition of the particles on air quality. A very few studies have addressed the important and more specific question of the efficiency of aerosol particle deposition in the respiratory tract after inhalation and the factors controlling the deposition efficiency. Inhaled particles deposit along respiratory tract mainly by diffusion, sedimentation and impaction. The primary mechanism by which the particles deposit depends on size of the particles, and location of deposition along the respiratory tract^15–17^. For small particles of size less than 0.5 μm, deposition is mainly by diffusion. Deposition by diffusion occurs often in the alveolar interstitium (AI) due to smaller airways. By the gravitational force exerted on the particles, particles great than about 0.5 μm deposit principally by sedimentation in AI and the part of the tracheobronchial airway (TB) close to AI. Impaction becomes dominant mechanism by which particles larger than 1 μm deposit in TB. More details about particle deposition mechanism can be found in Hussain *et al*., and Varghese *et al*.^16,17^.

A previous study by Kajino *et al*. indicated that the aerosol mixing state affected the efficiency of aerosol deposition in the human respiratory tract^18^. Aged soot particles undergo hygroscopic growth depending on their hygroscopicity, and as they grow in size, they deposit less efficiently than particles that did not undergo such hygroscopic growth. Building upon the study by Kajino *et al*., here, we investigate the regional deposition efficiency of an aerosol population containing particles with a range of mixing states and sizes^18^. While the aerosol population was assumed to have a log-normal size distribution and a constant hygroscopicity value in Kajino *et al*.^18^, here, we examine specifically for an aerosol population undergoing the atmospheric aging process in an urban plume environment, how the evolving aerosol particles sizes, mixing state and hence hygroscopicities impact the regional deposition efficiencies through particle hygroscopic growth. The term regional deposition refers to the deposition in different regions along the human respiratory tract. Aerosol aging means the evolution of the mixing of chemical species within each individual particle in an aerosol population. Aerosol aging occurs in the atmosphere due to Brownian coagulation among particles, condensation of gaseous phase chemicals on the particles and heterogeneous chemical reactions on the particles.

**Figure 1:**
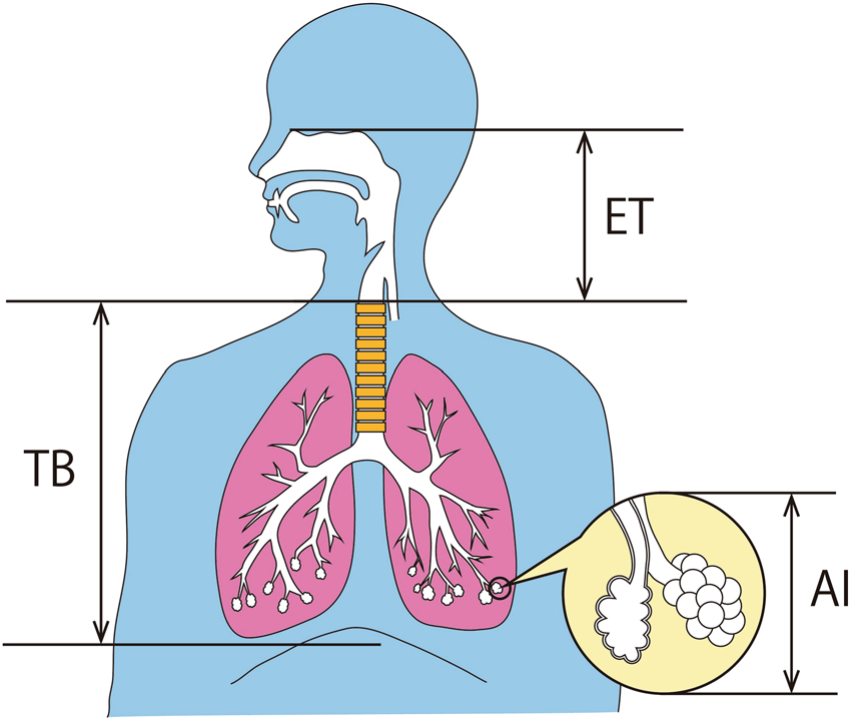
The locations of the extrathoracic airway (ET), the tracheobronchial airway (TB), and the alveolar interstitium (AI) along the human respiratory tract. The illustration of human respiratory tract here is produced by Ms. Yuri Tachikawa of the Meteorological Research Institute (MRI).

Using the aerosol particle-resolved model PartMC-MOSAIC^19^ together with the regional deposition model of Kajino *et al*.^18^, this study is the first to determine the regional deposition efficiency of inhaled aerosol particles atmospherically aged to various extents. Based on detailed particle-resolved simulations of the aerosol physico-chemical properties, the deposition efficiency of inhaled particles at three locations along the human respiratory tract, namely, the extrathoracic airway (ET), the tracheobronchial airway (TB), and the alveolar interstitium (AI), are calculated. The locations of ET, TB and AI are illustrated in Fig. 1. In addition, this study employs the recently developed aerosol mixing state metric to quantify the impact of the mixing state on the particle deposition efficiency in the human respiratory tract^20^. The results offer insight into how the aerosol mixing state impacts air quality and human health in atmospherically relevant urban environments.

The following result section presents simulation results and analyzes the impact of the aerosol mixing state and its evolution on the deposition of inhaled particles along the respiratory tract. Brief descriptions of the particle-resolved aerosol model, the deposition model and the simulations performed in the current study are given in the methodology section.

## Results

In this section, the evolution of the mixing state simulated by PartMC-MOSAIC is presented first, followed by the mixing-state-dependent hygroscopic growth of the particles computed by the regional deposition model. Then, we present and discuss the regional deposition efficiency computed based on the particle-resolved mixing state and hygroscopic growth. Finally, we determine the impact of the aerosol mixing state on the deposition efficiency by quantifying the error in the deposition efficiency due to the simplified aerosol mixing state representation.

### Aerosol mixing state evolution simulated by PartMC-MOSAIC

**Figure 2:**
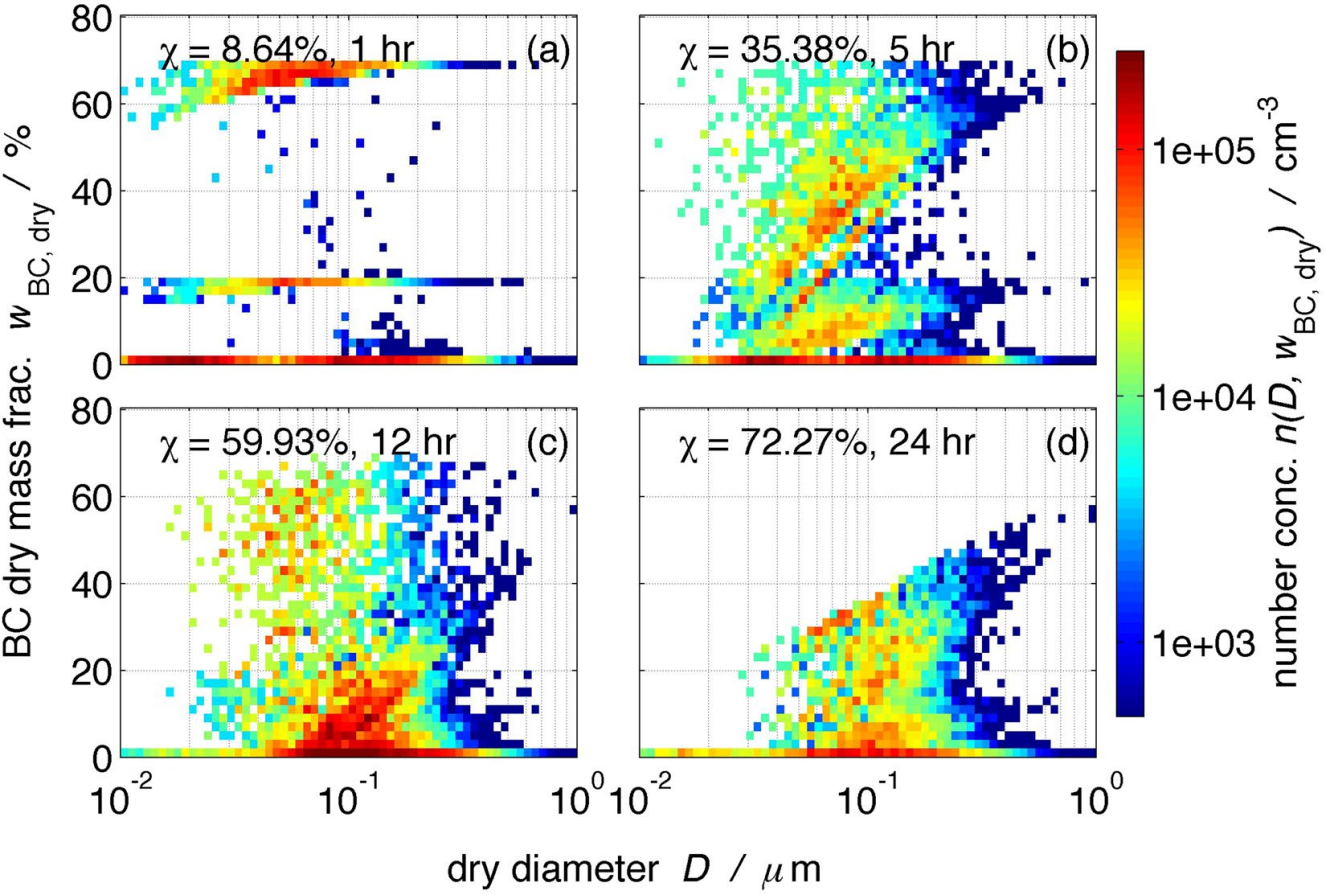
The aerosol mixing state of the urban aerosol populations simulated by the particle-resolved aerosol model PartMC-MOSAIC, which are then input to the human respiratory tract deposition model. The aerosol mixing state is presented in terms of number concentration as a function of dry diameter and black carbon (BC) dry mass fraction. The four panels show the temporal evolution of the aerosol mixing state. The time and aerosol mixing index χ of each snapshot are indicated in each panel.

The aerosol mixing state evolution in an urban plume scenario simulated by PartMC-MOSAIC is shown in Fig. 2^21^. The number concentration, denoted by *n*(*D*_dry_, *w*_BC_), is defined in the same way as Equation 13 in Ching *et al*.^21^. The BC mass fraction, *w*_BC_ is simply defined as the ratio of the BC mass to the total dry mass of the aerosol particles. In each panel of Fig. 2, the aerosol mixing state index χ is given at the top left and would be discussed in detail in the subsection of aerosol mixing state index. At hour 1, the three straight lines in Fig. 2a correspond to soot particles from diesel engine combustion (*w*_BC_ = 70%), gasoline engine combustion (*w*_BC_ = 20%) and meat cooking (*w*_BC_ = 0%). In addition, some ammonium sulfate background aerosols are present (*w*_BC_ = 0.8%). Gradually, the *w*_BC_ of the particles decrease due to Brownian coagulation among particles and the condensation of non-BC gaseous precursors on the particles. Consequently, intermediate *w*_BC_ values are developed. Furthermore, the diagonal patterns in Fig. 2b–d, running from small diameter at low *w*_BC_ to large diameter at high *w*_BC_, are due to the faster decrease in the *w*_BC_ of smaller particles by condensation than that of larger particles. Since the air parcel under simulation has experienced typical aerosol emissions until hour 12, the horizontal linear pattern at *w*_BC_ = 20% and *w*_BC_ = 70% in Fig. 2a which represent fresh emissions, disappear after hour 12, for example, in Fig. 2d (hour 24). At hour 36 (figure not shown), most particles have *w*_BC_ < 40%. During the course of aging, the hygroscopicity and size of the particles increase, while *w*_BC_ decreases.

### Hygroscopic growth of aerosol simulated by regional deposition model

The hygroscopic growth of particles along the human respiratory tract was calculated by the regional deposition model, following the description in the methodology section and Equation 3^18^. Since it was discussed in detail in the work of Kajino *et al*.^18^, we only include the aerosol hygroscopic growth calculated for particles of two selected dry diameters in Figure S1 for reference. In this study, the environment along the respiratory tract is saturated with water vapor (relative humidity of 99.5%) and set at normal body temperature (37 °C). Figure S2 depicts the sensitivity of the aerosol deposition efficiency, *e*, at the ET, TB and AI as a function of dry diameter and hygroscopicity of the particles simulated by the deposition model for an adult male during light exercise using the nose to breathe. We found that larger particles deposit less efficiently than smaller ones in the TB and AI. The deposition efficiency in ET, TB, AI is more sensitive to the particle dry diameter than the hygroscopicity. Therefore, it is critical to accurately compute the particle hygroscopic growth along the respiratory tract after inhalation to evaluate the aerosol deposition along the respiratory tract.

### Aerosol deposition along human respiratory tract

#### Gross deposition efficiency

With detailed particle-level information of the size and mixing state simulated by PartMC-MOSAIC, the particle hygroscopicity and associated hygroscopic particle growth, which evolve during the course of the aerosol aging process, can be readily computed. Here, we define the gross deposition efficiency, *F*, of soot for the whole aerosol population based on the deposition efficiency calculated for individual particles as follows,

1 $$F=\frac{{\sum }_{i=1}^{N}\,{e}_{i}({D}_{i},{\kappa }_{i})\,{m}_{{\rm{soot}},i}}{{\sum }_{i=1}^{N}\,{m}_{{\rm{soot}},i}}$$

where *e*_*i*_ (*D*_*i*_, *k*_*i*_) is the deposition efficiency of particle *i* with diameter *D*_*i*_ and hygroscopicity *k*_*i*_ and ${m}_{{\rm{soot}},i}$ is the mass concentration of soot contained in particle *i*.

Here, we define the mass of soot simply as the sum of the masses of BC and primary organic carbon. It should be noted that *e*_*i*_ is a function of time since both *D*_*i*_ and ${\kappa }_{i}$ are functions of time in the aerosol aging process. Besides, *e*_*i*_ is also a function of age, gender, mode of inhalation and human activity, as well as the location of deposition in the respiratory tract.

**Figure 3:**
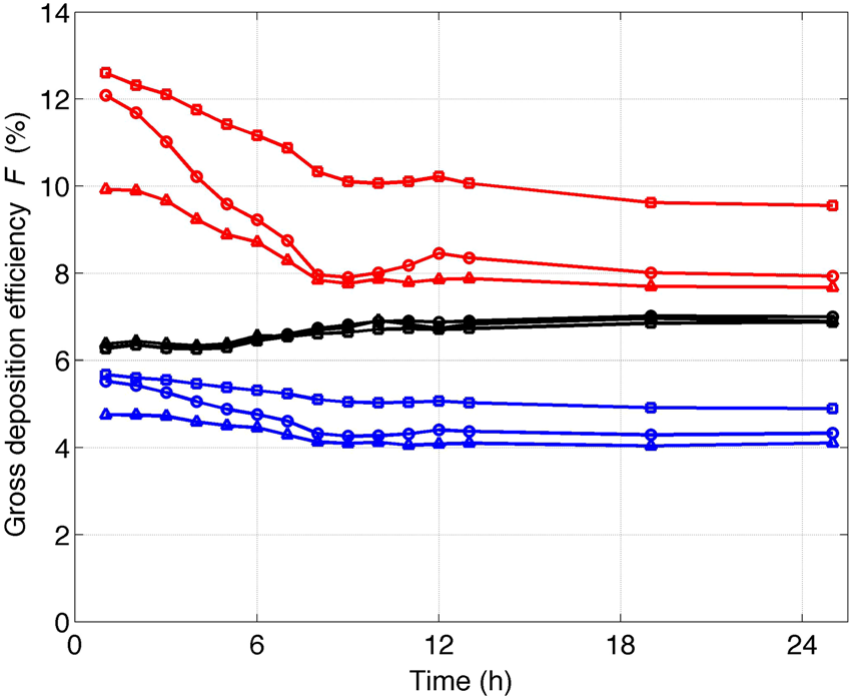
The gross deposition efficiency *F* of an adult male during light exercise with nasal inhalation verses time. Hour 0 corresponds to 6:00 in the morning. The three colors indicate the three locations in the human respiratory tract, AI (red), TB (blue) and ET (black). Three sets of simulations were performed, particle-resolved simulation (circle), composition-averaged simulation (triangle), and zero-hygroscopicity simulation (square). All simulations were performed at the relative humidity of 50%.

Figure 3 shows the evolution of *F* for the first 25 hours over a 48-hour simulation in the AI (red), TB (blue) and ET (black) of an adult male during light exercise with nasal inhalation. The open circles indicate the particle-resolved simulations at initial environmental relative humidity of 50%. *F* in the AI gradually decreases from approximately 12.1% at hour 1 to 7.9% at hour 24, *F* in the TB decreases from 5.5% to 4.3%, and *F* in the ET stays approximately between 6.3% and 7%. At hour 1, the aerosol population contains mostly freshly emitted soot particles from diesel and gasoline engine combustion, which are hydrophobic, while at later times, the particles become gradually more hygroscopic through the aging process. Since the particles experience a water-vapor-saturated environment below the larynx (AI and TB), they grow to a large size and deposit less efficiently in the AI and TB, which is consistent with Figure S2. In contrast, in the ET, the gross deposition efficiency *F* does not change significantly with time. This is due to the fact that the equilibrium wet diameter to which the particles grow in the ET at relative humidity of 50% is not sensitive to the particle hygroscopicity. Hence, the evolution of the mixing state does not lead to significant evolution in the deposition efficiency *F*.

To demonstrate the effect of particle hygroscopic growth on the gross deposition efficiency along the respiratory tract, we repeated the deposition simulations for all the aerosol populations, but with the particle hygroscopicity set to 0 while keeping the same particle dry diameters. We found that assuming completely hydrophobic particles leads to overestimation of the deposition efficiency of inhaled particles in the AI and TB. In Fig. 3, comparing the results of zero-hygroscopicity simulation (squares) with those of particle-resolved simulation (circles), we see that the overestimation (the absolute difference between the squares and circles) is between 2.4% and 0.5% for the AI and between 0.78% and 0.15% for the TB. This overestimation is attributed to the neglect of the hygroscopic growth of the particles, which gives rise to more efficient deposition, and it is consistently shown in Figure S2 that small particles deposit more efficiently in the AI and TB. However, there is no significant difference in the deposition in the ET between the two sets of simulations (black circles and black squares). The relative humidity in the ET equals the ambient relative humidity, which is 50% in this case. When calculating the deposition efficiency, the particles are assumed to grow to the equilibrium wet diameter at the relative humidity in the ET. Since the equilibrium wet diameter is not sensitive to the hygroscopicity at the relative humidity of 50%, the deposition efficiency *F* is not significantly different between the two sets of simulations.

Since particle hygroscopic growth affects the aerosol deposition efficiency and depends on the aerosol mixing state, to examine the impact of the aerosol mixing state on the regional deposition efficiency, we simplified the aerosol mixing state representation input to the regional deposition model by averaging the size and composition of the aerosol particles. The detailed mathematical procedure for the averaging can be found in the appendix of Ching *et al*.^21^. In brief, for each of the PartMC-MOSAIC-simulated mixing-state-resolved aerosol populations, we performed a size-resolved averaging procedure by first sorting the particles into twenty five logarithmically spaced size bins from 0.1 nm to 10 μm according to the particle dry diameter. Then, all particles within each size bin are assigned the bin-averaged particle volume and bin-averaged composition. For simplicity, the aerosol population that underwent this averaging procedure is hereafter referred to as the averaged population. We input these averaged aerosol populations to the deposition model to calculate the gross deposition efficiency, *F*, of soot, which is shown by triangles in Fig. 3. We found that simplifying the mixing state generally leads to the underestimation of the deposition efficiency (the absolute difference between the triangles and circles) in the AI and TB by 0.1–2.2% for the environmental scenario investigated here over 25 hours. It is important to note that the number and mass concentration are conserved in this averaging procedure. The difference in the simulations results using the particle-resolved populations (circles) and the averaged populations (triangles) are attributed to the modified aerosol mixing state and size representation input to the model and will be discussed in detail in the subsection of impact of mixing state on deposition efficiency.

A paired t-test is performed and shows that the differences in *F* between (1) the particle-resolved populations and the zero-hygroscopicity populations; and (2) the particle-resolved populations and the averaged populations are statistically significant at 99% for most of the scenarios considered in this study. Tables summarizing the results are given in Tables S6–S9.

#### Aerosol mixing state index

To quantify and examine the impact of the aerosol mixing state on the aerosol deposition efficiency, we quantify the aerosol mixing state of the aerosol population using the metric developed by Riemer and West^20^. The aerosol mixing state index is derived from information theoric entropy measures. Readers are referred to the work of Riemer and West for details^20^. Briefly, the mixing state index, denoted by χ, is calculated according to (1) the mass fraction of each chemical species contained in each individual particle and (2) the total bulk mass fraction of each chemical species contained in the aerosol population. The computed mixing state index ranges from 0% to 100%, where 0% indicates a completely externally mixed aerosol population and 100% corresponds to a completely internally mixed population. In the atmosphere, aerosol populations rarely exist as completely internally or externally mixed populations, whereas they usually exist in certain intermediate states between the two extremes. Thus, the mixing state index χ provides a quantitative measure to represent the mixing state of an atmospheric aerosol population more appropriately than conventional qualitative descriptions found prevalently in the literature. Besides, the aerosol mixing state index χ serves as a comprehensible metric against which the error in the deposition efficiency can be quantified to elucidate the impact of the mixing state on aerosol deposition along the human respiratory tract. This will be further discussed in the next subsection. The same aerosol mixing state index has been applied to analyze the error in climate-relevant quantities due to the simplified mixing state representation, for example, the error in the cloud condensation nuclei concentration in Ching *et al*.^22^.

#### Impact of mixing state on deposition efficiency

**Figure 4:**
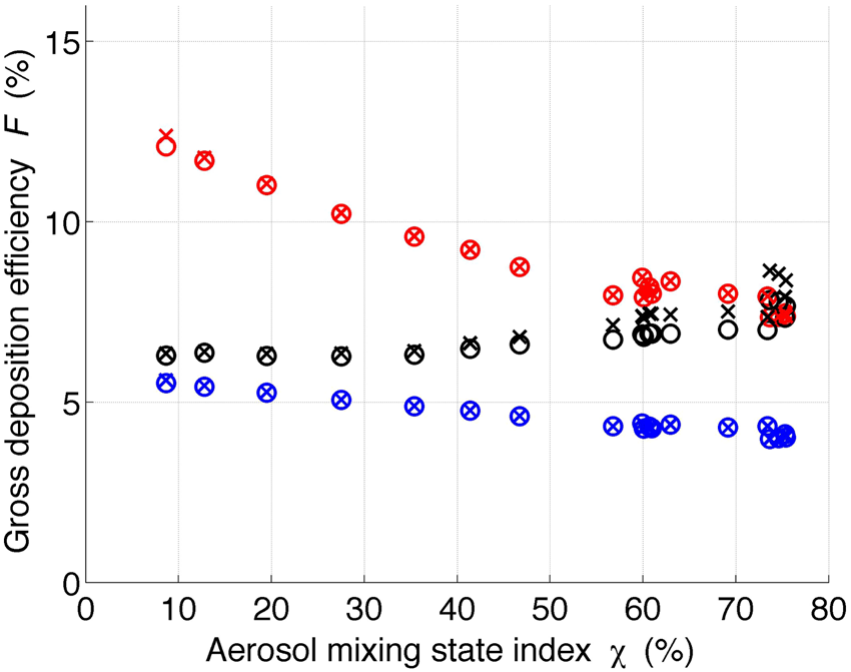
The gross deposition efficiency *F* of an adult male during light exercise with nasal inhalation verses aerosol mixing state index χ. The three colors indicate the three locations in the human respiratory tract, AI (red), TB (blue) and ET (black). The circles and crosses represent the initial environmental relative humidities of 50% and 80%, respectively, input to the deposition model.

Over the 48-hour simulation, the mixing state of the aerosol population constantly evolves through the process of condensation and coagulation and is modulated by emission and the exchange between the plume and environment due to dilution. One analysis perspective is the temporal perspective, which is presented in Fig. 3. This perspective illustrates, along the time dimension, how the evolution of the mixing state in an urban environment impacts aerosol deposition in the respiratory tract. Another analysis perspective is to apply the mixing state index χ. This examines the deposition of aerosol populations with various mixing extents, regardless of the temporal history of the aerosol populations. This perspective clearly elucidates the relationship between the deposition efficiency and aerosol mixing state and is presented in Fig. 4.

In addition, to examine the influence of relative humidity on the particle deposition, we also performed simulations at initial environmental relative humidity of 80%, apart from 50%. The simulated deposition efficiency *F* of particle-resolved populations is plotted against the aerosol mixing state index χ in Fig. 4 to illustrate the relationship between the two quantities. Since we focus on the deposition of soot particles, which are composed of BC and POA (Table S2), when calculating χ, we consider BC and POA as one surrogate species, and all remaining aerosol species are considered as a second surrogate species. Then, χ is calculated based on the mass fractions of these two surrogate species. For clarity, the results of 19 populations that encompass a wide range of mixing state index χ values were selected from the 48-hour PartMC-MOSAIC simulation and are presented in Fig. 4.

In Fig. 4, when the populations become more internally mixed (large χ value), the $F$ in the AI (red) and TB (blue) decrease. This is due to the increase in the particle hygroscopicity and the associated hygroscopic growth, which was explained earlier. Besides, the $F$ in the AI and TB at initial relative humidities of 50% (circles) and 80% (squares) do not significantly differ. This is because the deposition in the AI and TB is computed assuming that the particles grow in an environment saturated with water vapor at body temperature (37 °C) and relative humidity of 99.5%, regardless of the initial relative humidity input to the model. On the other hand, the $F$ in the ET for initial relative humidity of 50% does not vary as notably with χ as that at relative humidity of 80%. This originates from same reason discussed above: the lower sensitivity of the wet equilibrium diameter to the aerosol mixing state at lower relative humidity.

The results in Figs 3 and 4 are based on the aerosol deposition efficiency in an adult male during light exercise with nasal inhalation. In addition, we performed simulations for 10-year old child. The deposition simulations for both adult and 10-year old child were also performed during heavy exercise and rest at initial relative humidity of 50% with nasal inhalation apart from light exercise. The results from temporal perspective and mixing state index χ perspective are shown in Figures S4 and S5 respectively. The middle right panel of Figures S4 and S5 are the same as Figs 3 and 4 respectively. In Figure S4, it is seen that for both adult male and 10-year old child, the deposition efficiency *F* in the ET (black) is larger during heavy exercise than during light exercise, which is in turn larger than body at rest. The reverse of this order holds for AI (red). Comparing between adult male and 10-year old child, the deposition efficiency *F* in the Al (red) is higher for 10-year old child than for adult male during rest; while during heavy exercise, the deposition efficiency *F* in the ET (black) is higher for adult male than for 10-year old child. For light exercise, the deposition efficiency *F* in the AI, TB and ET for 10-year old child and adult male demonstrates similar patterns and magnitudes. In Figure S5, it is seen that similar patterns of deposition efficiency *F* in the AI, TB and ET versus mixing state index χ appear between adult male and 10-year old child during heavy exercise, light exercise and rest. From many previous studies, inhaled particle deposition efficiency was found to depend on physical activities. Londahl *et al*. conducted measurements of respiratory tract deposition for about 30 adults^23^, and found that difference in deposition fractions during exercise and rest was less than 3%, whereas, measurements by Daigle *et al*. showed that deposition fraction of ultrafine particles was higher in human during exercise than at rest by 17%^24^. Oravisjarvi *et al*. performed model simulations for adult male, female, children of 10 years old and 5 years old during sleeping, sitting, light and heavy exercise^25^. They indicated that more particles deposited in males than in females, and children have less particle deposition than adult males and females. Physical exercise leads to more particle deposition than staying at rest.

In short, the results shown in Figure S4 and S5 indicate that, based on particle-resolved simulations, deposition efficiency depends on the location of deposition (Al, TB and ET) and aerosol mixing state, alongside with age, sex and activity level. The dependence of deposition efficiency on aforementioned factors agrees partly with what previous studies found. This could be due to the different simulation setups, model input (e.g. size distribution and composition of aerosol) and model treatment of aerosol mixing state. Further studies are necessary to improve the models simulating particle depositions so as to constrain the difference between the measurement and model simulations in future.

**Figure 5:**
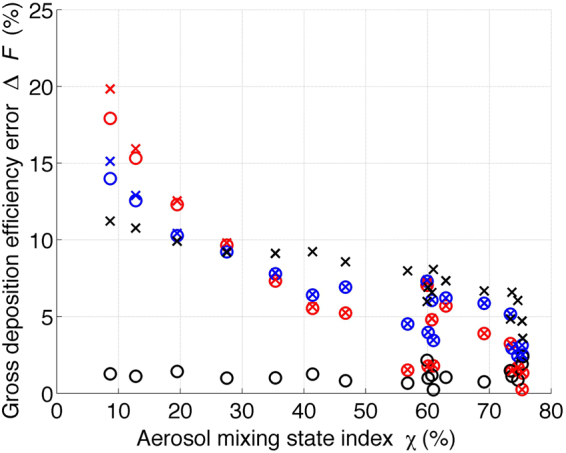
The error in the gross deposition efficiency Δ*F* of an adult male during light exercise with nasal inhalation verses the aerosol mixing state index χ. The three colors indicate the three locations in the human respiratory tract, AI (red), TB (blue) and ET (black). The circles and crosses represent the initial environmental relative humidities of 50% and 80%, respectively, input to the deposition model.

It was found that the aerosol mixing state impacts the aerosol deposition efficiency in the respiratory tract. However, many regional and global climate and meteorology models simplify the aerosol representation due to limited computational resources. Therefore, it is instructive to evaluate the discrepancy in the aerosol deposition efficiency caused by the simplified aerosol representation. Here, we follow the approach of Ching *et al*. by regarding the deposition efficiency calculated by the particle-resolved simulation as the benchmark^21,22,26^. We define the error in the deposition efficiency, Δ*F*, as the relative difference between the deposition efficiency calculated by PartMC-MOSAIC particle-resolved simulations, denoted by *F*, and that calculated by simulations using the averaged population (the subsection of gross deposition efficiency), denoted by $\bar{F}.$ The error quantifying framework can be expressed mathematically as

2 $${\rm{\Delta }}F\,(\chi )=\frac{\overline{F(\chi )}-F(\chi )}{F\,(\chi )}$$

where $\bar{F}(\chi )$ and $F(\chi )$ are the *F* of populations with a mixing state index of $\chi$. Figure 5 depicts the error ${\rm{\Delta }}F$ verses $\chi$ for the same 19 aerosol populations shown in Fig. 4. For convenient interpretation, we neglect the negative sign of ${\rm{\Delta }}F$ (according to Equation 2, a negative sign indicates underestimation by composition averaging procedure), and only the absolute magnitude ($|{\rm{\Delta }}F|$) is plotted in Fig. 5.

In Fig. 5, it is clear that ${\rm{\Delta }}F$ decreases when $\chi$ increases for the deposition efficiency in the AI (red) and TB (blue) at initial relative humidities of 50% and 80%. For more externally mixed populations ($\chi  < 10 \%$), the error is more than 14%, while for more internally mixed populations ($\chi  > 70 \%$), the error drops to 5% or less. The error ${\rm{\Delta }}F$ originated from the aerosol mixing state simplification can be understood as follows: simplifying the mixing state by averaging the particle size and composition for the urban plume scenario investigated in this study leads to overestimation of the hygroscopicity and therefore the associated hygroscopic growth. This results in underestimation of the deposition efficiency. For more internally mixed populations, represented by large $\chi$ values, the particles within the population have a more homogeneous composition. Therefore, the averaging procedure does not significantly modify the particle hygroscopicity and hence the associated hygroscopic growth and gives rise to a smaller ${\rm{\Delta }}F$ than that for populations with smaller $\chi$.

It is found that the error ${\rm{\Delta }}F$ in the AI (red) for initial environmental relative humidities of 50% and 80% do not significantly differ, and the same also holds for ${\rm{\Delta }}F$ in the TB (blue). The reason for this is that deposition in the AI and TB is computed regardless of the initial relative humidity input to the model, which is consistent with the results in Fig. 4 (red and blue crosses and circles). On the other hand, the deposition errors ${\rm{\Delta }}F$ in the ET (black) in Fig. 5 at relative humidities of 50% and 80% behave differently. This is because the deposition in the ET is calculated assuming the particles grow to the equilibrium diameter corresponding to the environmental relative humidity, which is the same as the initial relative humidity input to the model. Since the equilibrium wet diameter is more sensitive to particle hygroscopicity and therefore the mixing state, at higher relative humidity, the error ${\rm{\Delta }}F$ originated from the simplified aerosol mixing state is more remarkable at relative humidity of 80% (black crosses) than at relative humidity of 50% (black circles). Figure S6 shows the absolute magnitude of deposition error $(|{\rm{\Delta }}F|)\,$verses ${\rm{\chi }}$ for both adult and 10-year old child during light, heavy exercise and rest at initial relative humidity of 50%. The middle right panel of Figure S6 is the same as Fig. 5. In general, the magnitude of $(|{\rm{\Delta }}F|)$ decreases when the aerosol population becomes more internally-mixed (towards larger ${\rm{\chi }})$ for both deposition in the AI (red) and TB (blue) in all the six panels. The low sensitivity of $(|{\rm{\Delta }}F|)$ in the ET at relative humidity of 50% (black circles) to aerosol mixing state ${\rm{\chi }}$ holds for both adult and 10-year old child during light, heavy exercise and rest. This is due to the same reason discussed above for Fig. 5.

## Discussion

The evolution of the aerosol mixing state in the atmosphere plays a significant role in determining the impact of air quality on public health. Because the human respiratory tract is saturated with water vapor, the inhaled aerosols grow in size depending on their hygroscopicity and therefore mixing state. This study demonstrates that without considering mixing-state-dependent hygroscopic growth of particles leads to overestimation of deposition efficiency; whereas considering an average mixing state leads to underestimation of 5% to 20% of soot particle deposition efficiency in human alveoli. In addition, it is found that aged soot particles deposit less efficiently in the AI and TB due to hygroscopic growth along the respiratory tract. However, it is important to recognize that it has not been determined whether air plumes containing aged aerosol particles pose less of a threat to the human respiratory system. Other factors, including the absolute aerosol number concentration, aerosol surface area distribution, aerosol chemical composition, and meteorological and human physiological conditions, play remarkable roles in determining the harmfulness of polluted air. For example, hygroscopic growth and existence of certain chemical species may facilitate chemical reactions on aerosol surface or in bulk, which contribute to the particle toxicity towards human cells^27,28^. Particle growth could also increase the surface area for interactions between aerosols and cell membranes^29^.

Numerous epidemiology studies examined the exposure-risk function or the exposure-response function, which related the dose of PM2.5 and the risk of having cardiovascular and cardiopulmonary diseases (e.g. Pope *et al*.)^30^. To take into account the deposition efficiency in constructing the exposure-response function, it requires the consideration of mixing state, which entails the multidimensional parameter space of size-resolved composition and size distribution of PM. For example, it is expected that hygroscopic ammonium sulfate aerosols deposit less efficiently in human respiratory tract than hydrophobic soot particles emitted from the vehicles due to hygroscopic particle growth. However, there is a complication derived from the aerosol particles interactions with atmospheric gaseous components (condensation or evaporation) or among one another (coagulation), collectively known as aerosol aging, which modify the aerosol physico-chemical properties and therefore deposition efficiency. The deposition efficiency of an internally-mixed soot and sulfate particle would behave differently from either a pure soot or sulfate particle. Environmental tobacco smoke (ETS), a major human activity-related pollutant, consists of various gaseous phase and aerosol phase organic and inorganic chemical species. The sizes and mixing state of the ETS particles evolve in the surrounding environment and deposit differently in the respiratory tract from what soot and sulfate particles do^31^. It is noteworthy that in addition to modulating the deposition efficiency, mixing state has probably broad implications on particle toxicity and physical and biochemical interactions with human tissues after deposition. These impacts could further complicate the exposure-response function. This research indicates the potential importance of mixing state for constructing a more comprehensive and accurate exposure-response function.

Since industrial revolution, air quality has been a great concern from time to time. In particular, in recent years, the public has expressed increasing awareness of air quality and keenness for clean air in the midst of economic development. In the theme of sustainable development in the anthropocene, further laboratory experiments, field campaign measurements, and model simulations ranging from process level to regional atmospheric scales are necessary to elucidate the convoluted interactions between meteorology, atmospheric chemistry, anthropogenic activities and human physiological response.

## Methodology

### Particle-resolved model simulating aerosol dynamics and chemistry

Riemer *et al*. developed a particle-resolved aerosol model^19^, PartMC-MOSAIC (Particle Monte Carlo-Model for Simulating Aerosol Interactions and Chemistry), that simulates the evolution of the composition of individual particles in an atmospheric aerosol population. For details of PartMC-MOSAIC model, readers are recommended to refer to the work of Riemer *et al*.^19^, which gives a comprehensive description of the model, including the governing equations and numerical algorithms of PartMC-MOSAIC. Here, we briefly introduce the model.

PartMC-MOSAIC model is composed of two components, PartMC and MOSAIC.

PartMC simulates the evolution of the aerosol composition in a well-mixed computational volume in the atmosphere due to Brownian coagulation among particles, the mixing of particles between the background atmosphere and the volume and the particle emission into the volume using the Monte Carlo method. To tackle the aerosol chemistry, PartMC is coupled to the aerosol chemistry module MOSAIC^32^, which was implemented in the widely used regional weather research and forecast and chemistry model, WRF-Chem^33,34^.

MOSAIC models gas-phase photochemistry using the carbon bond mechanism CBM-Z^35^, which considers 142 reactions. Along with CBM-Z mechanism, MOSAIC consists of three modules: (1) the Multicomponent Taylor Expansion Method (MTEM)^36^, (2) the Multicomponent Equilibrium Solver for Aerosols (MESA)^37^, and (3) the Adaptive Step Time-split Euler Method (ASTEM)^32^. These modules are used to estimate the activity coefficients of electrolytes and ions in aqueous solutions^36^, solve for intraparticle solid-liquid partitioning^37^, and solve for dynamic gas-particle partitioning, respectively, in a deterministic manner^32^, in contrast to the stochastic modeling approach of PartMC. MOSAIC incorporates 77 gaseous species and 19 aerosol species that are locally and globally important. The aerosol species include SO_4_, NO_3_, Cl, CO_3_, MSA (methanesulfonic acid), NH_4_, Na, Ca, other inorganic mass (such as SiO_2_, metal oxides, and other unmeasured or unknown inorganic species present in aerosols), black carbon (BC), primary organic aerosol (POA) and secondary organic aerosol (SOA). SOA is treated by the Secondary Organic Aerosol Model (SORGAM) scheme^38^. Four aerosol species represent SOA derived from the oxidation of anthropogenic volatile organic compound (VOC) precursors (ARO1, ARO2, ALK1, and OLE1), and four represent SOA derived from biogenic VOC precursors (LIM1, LIM2, API1, and API2). For a complete description of MOSAIC, readers are referred to Zaveri *et al*.^32^.

PartMC-MOSAIC model output contains detailed physico-chemical quantities of individual particles. Among these quantities, an important one is the mass of each aerosol species comprising individual particles. Based on this, the size and mass of individual particles, bulk mass concentration of any aerosol species, aerosol number concentration and size distribution can be conveniently computed. Furthermore, climate-relevant properties, such as cloud condensation nuclei concentration and optical properties, can be calculated whenever needed.

PartMC-MOSAIC is a unique tool to investigate the aerosol mixing state evolution at particle level to further our understanding of the aging process and the associated impacts on climate-relevant properties. Because PartMC-MOSAIC imposes no intrinsic assumption about the mixing state of the aerosol population and is free of numerical diffusion^19^, it has been used to benchmark the performance of models with simplified aerosol representation. Specifically, PartMC-MOSAIC has been applied to investigate the aging of BC-containing particles and the associated errors in cloud microphysical and aerosol optical properties due to simplified mixing state representations^21,22,26,39^. In addition, Tian *et al*. used PartMC-MOSAIC to study the evolution of the aerosol composition in ship plume and chamber environments^40,41^, while Fierce *et al*. studied the influence of BC particle aging on climate and the environmental dependence of the aging time scale^42^.

### Regional deposition model considering particle hygroscopic growth in the body

In this study, following a United States-Environmental Protection Agency (US-EPA) report^43^, the human respiratory tract was classified into three regions: ET (extrathoracic airway), TB (tracheobronchial airway), and AI (alveolar interstitium). The Multiple Path Particle Dosimetry Model (MPPD ver 2.11; U.S. Applied Research Associates (ARA) Inc.) was applied to calculate the deposition efficiency to each region depending on the particle size, density and human conditions (age, and sex)^44,45^. Other necessary parameters, such as respiratory volume and breathing frequency, were taken from an *ICRP* publication^15^.

Because the respiratory tract below the larynx (bronchi and alveolar) is saturated with water vapor, hygroscopic growth inside the body must be taken into account; however, conventional models, such as the MPPD and ICRP models, do not consider this factor. Combustion-generated soot, which carries toxic substances, such as polycyclic aromatic hydrocarbons, is generally hydrophobic when emitted (fresh soot), but condensation of the hygroscopic components on soot during long-range transport makes the soot particles larger and more hygroscopic (aged soot), and thus, the deposition in the lung (AI) decreases. Given the typical size and hygroscopicity of fresh and aged soot, the lung deposition of fresh soot is approximately 70% greater than that of aged soot, estimated by simply considering the hygroscopic growth of aerosols in the human respiratory tract^18^. The model used here is the same as that of Kajino *et al*. but is described again here since that paper is written in Japanese^18^.

The hygroscopic growth of aerosol particle *i* in the body was calculated as follows:

3 $$\frac{d{L}_{i}}{dt}=\frac{\pi }{4}\alpha \bar{c}{D}_{i}^{2}f({D}_{i},\,\alpha ){n}_{i}{\rho }_{a}({Q}_{v,sat}-{Q}_{v,s,i})$$

where ${L}_{i},\,{n}_{i},\,\,{\rm{and}}\,{Q}_{v,s,i}$ are the water content (kg m^−3^), number density (m^−3^), and equilibrium vapor mixing ratio at the aerosol surface (kg kg^−1^), which is a function of size and hygroscopicity. ${Q}_{v,s,sat}$ is the water vapor mixing ratio (kg kg^−1^) at 37 °C (body temperature) and 99.5% relative humidity^46^. ${\rho }_{a}$ is the air density (kg m^−3^), $\bar{c}$ is the molecular speed of water vapor (m s^−1^), and $\alpha$ is the mass accommodation coefficient, which is defined as unity^47^. *D*_*i*_ is the wet diameter of particle *i*. For the correction factor of the transition regime *f*, the work of Fuchs and Sutugin was used^48^. Inhaled aerosols do not immediately reach the equilibrium diameter. The hygroscopic growth rate of an aerosol particle depends on its size, its hygroscopicity, the ambient air conditions (namely, air temperature and humidity) and the residence time in the body. We assumed the residence time to be four seconds^46^. Equation 3 was integrated for four seconds to obtain the wet diameter in TB and AI. An aerosol particle with 100 nm diameter swells to the equilibrium diameter in a few seconds, whereas it takes about more than a few minutes for particles greater than 1 μm in diameter (see Fig. 2 in Kajino *et al.*^18^ or Figure S1 in the supporting information). In fact, the particle hygroscopic growth inside the body is very insensitive to ambient air conditions. However, it substantially affects the deposition in ET, because the wet diameter of aerosols above the larynx are assumed to be equilibrated with the ambient air conditions.

### Simulation setup

We applied PartMC-MOSAIC to simulate the evolution of the aerosol size and chemical composition in an idealized urban plume environment over 48 hours. The idealized urban plume scenario under investigation is the same as that described in Zaveri *et al*. and is derived from an air quality study in Southern California in Zaveri *et al*.^32,39^. The same urban plume scenario served as a case study in Zaveri *et al*. and Ching *et al*. to examine the aerosol aging phenomenon and its relationship with several climate-relevant quantities^21,22,26,39,49^. For further details of the urban plume scenario, readers are suggested to refer to Zaveri *et al*.^32,39^. We briefly describe the urban plume environment in the following paragraph.

In the urban plume scenario (Figure S3 in the supporting information), the air parcel under simulation is assumed to travel over an urban area for the first 12 hours starting from 6 am in the morning, receiving aerosol emissions from meat cooking sources, diesel engine and gasoline engine vehicles and gaseous emissions, including NOx, SO_2_, CO, and VOCs. In addition, the air plume exchanges trace gases and aerosols with background atmosphere over the entire 48-hour period. The background air contains 50 ppbv ozone and other trace gaseous species as well as internally mixed particles composed of ammonium sulfate, SOA and BC^49^. In short, the total mass concentration in the simulated urban plume increases for the first 12 hours, due to the dominance of emission over dilution with background air. Furthermore, the aerosol number concentration increases due to the dominance of emission over dilution with background air and coagulations among particles. After the first 12 hours, the air parcel is assumed to leave the urban area and remain in the nocturnal residual layer over ocean for the remaining time. The mass concentrations of BC and POA and particle number concentration decrease because the urban plume does not receive any emission after the 12^th^ hour and undergoes constant dilution with the background atmosphere. The mass concentrations of SOA and ammonium nitrate are governed by the balance between photochemistry and dilution. The size distributions of the initial, background and emitted aerosol populations prescribed in the model are given in Tables S1 and S2, and the initial, background and emitted trace gas concentrations are given in Tables S3 and S4.

From the PartMC-MOSAIC simulations, we obtained the particle-resolved model output containing the composition of individual particles in the urban plume air parcel. Based on this, the hygroscopicity, κ, of an individual particle was computed using the volume weighted rule^50^. The hygroscopicity of each particle equals the volume weighted mean of the hygroscopicities of the aerosol species contained in that particle. Table S5 provides the hygroscopicities of the aerosol species considered here. The hygroscopicities and dry diameters of the particles are then input to the regional deposition model to calculate the hygroscopic growth of individual particles and the associated deposition efficiency at three locations along the human respiratory tract, namely, the extrathoracic airway, the tracheobronchial airway, and the alveolar interstitium, abbreviated to ET, TB and AI, respectively.

### Data availability

Model output data can be obtained by contacting Joseph Ching (jching@mri-jma.go.jp).

## Electronic supplementary material

Supplementary Information
