# Supplementary material for: Aerosol mixing state matters for particles deposition in human respiratory system
Source: Sci Rep. 2018 Jun 11;8:8864. doi: 10.1038/s41598-018-27156-z (PMC5995922; doi:10.1038/s41598-018-27156-z)
Supplement: Supplementary file 1 — Supplementary Information [file 41598_2018_27156_MOESM1_ESM.pdf]

Supporting information for the manuscript entitled

Aerosol mixing state matters for particles deposition in human respiratory system

Joseph Ching[1] and Mizuo Kajino[1,2]

[1]Meteorological Research Institute, Japan Meteorological Agency, 1-1 Nagamine, Tsukuba, Ibaraki 305-0052, Japan

[2]Faculty of Life and Environmental Sciences, University of Tsukuba, 1-1-1 Tennodai, Tsukuba, Ibaraki 305-8577, Japan

This supporting information consists of 14 pages. Figures S1-S6 and tables S1-S9 are included.

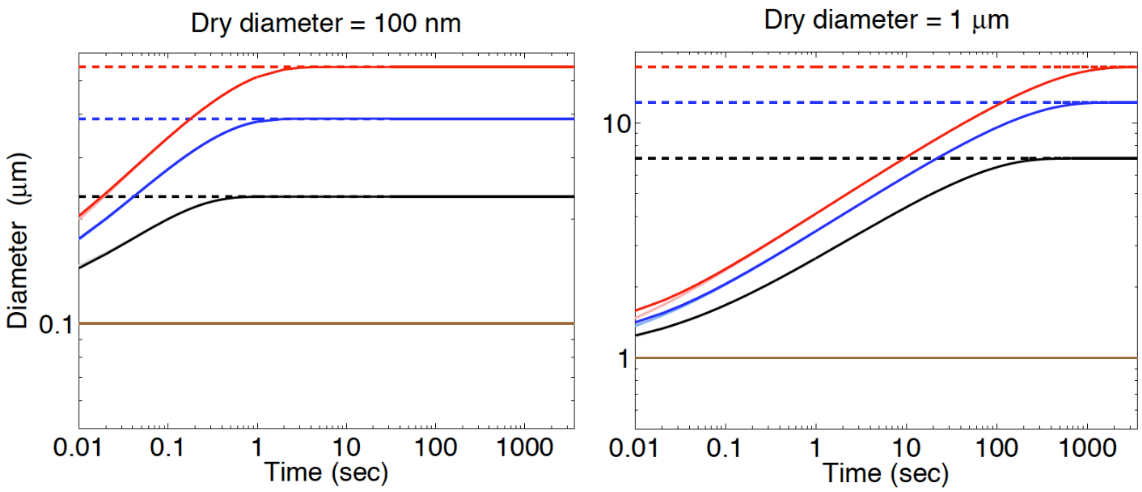

Figure S1. The hygroscopic growth of particles of 100 nm (left panel) and 1 μm (right panel) in diameter verses time. The three colors indicate the hygroscopicities of the particles under simulations with initial relative humidity of 70%, black (hygroscopicity parameter kappa = 0.3), blue (0.6) and red (1). The grey, light blue and pink lines represent respectively the particle growth of the same hygroscopicity indicated by their deeper color counterparts in the simulations with initial relative humidity of 30%. The dashed lines indicate the wet diameters to which the particles grow when they are in equilibrium with the relative humidity of 100%. The brown solid lines at 100 nm (left panel) and 1 μm (right panel) indicate the dry diameters for reference.

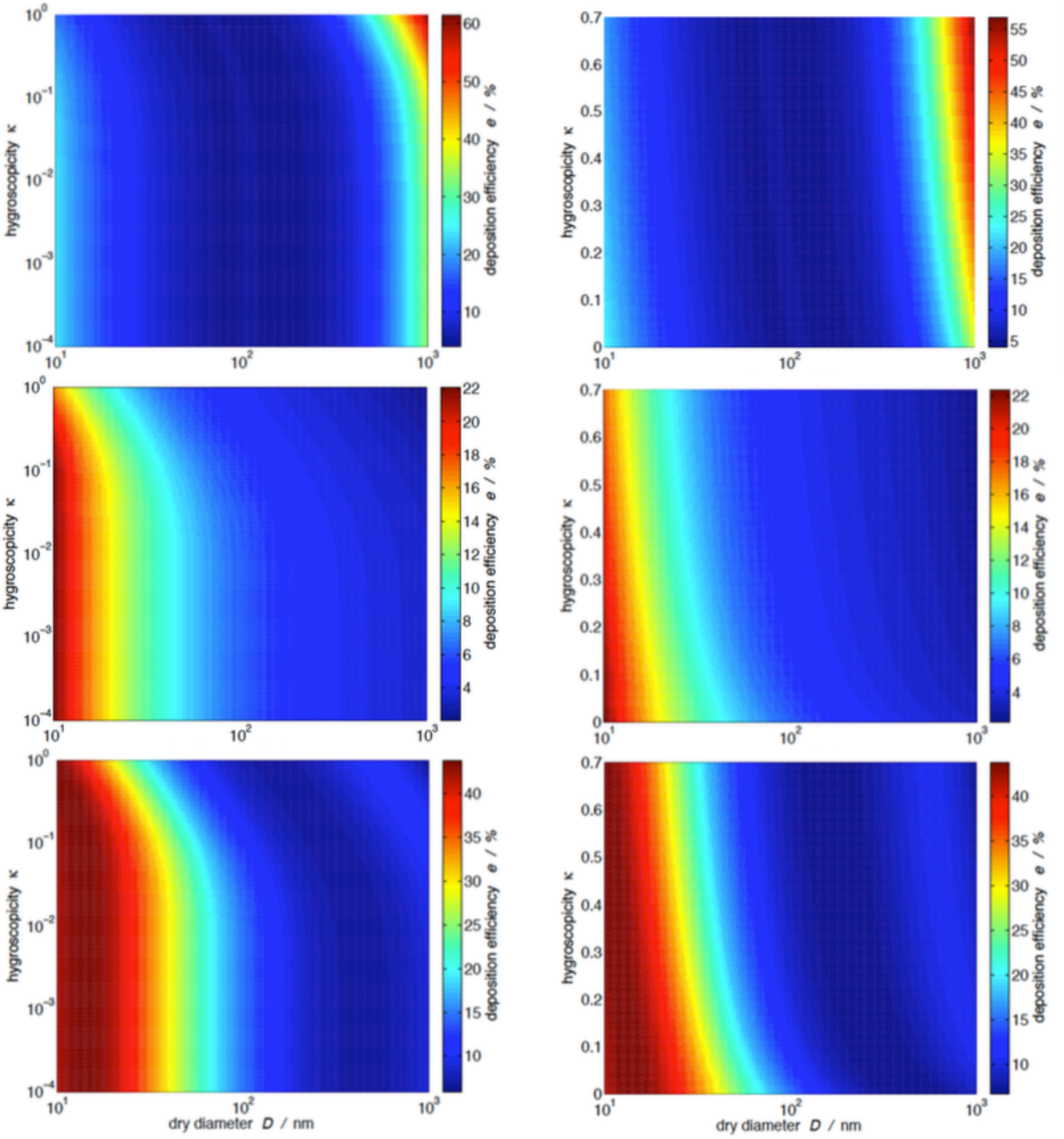

Figure S2. The deposition efficiency *e* as a function of dry diameter and hygroscopicity of the particles. The top, middle and bottom panels show the deposition efficiency at ET (extrathoracic airway), TB (tracheobronchial airway), and AI (alveolar interstitum) respectively, for an adult male in light exercise and breathing with nose. The left and right columns are plotted in logarithmic and linear scale for hygroscopicity respectively.

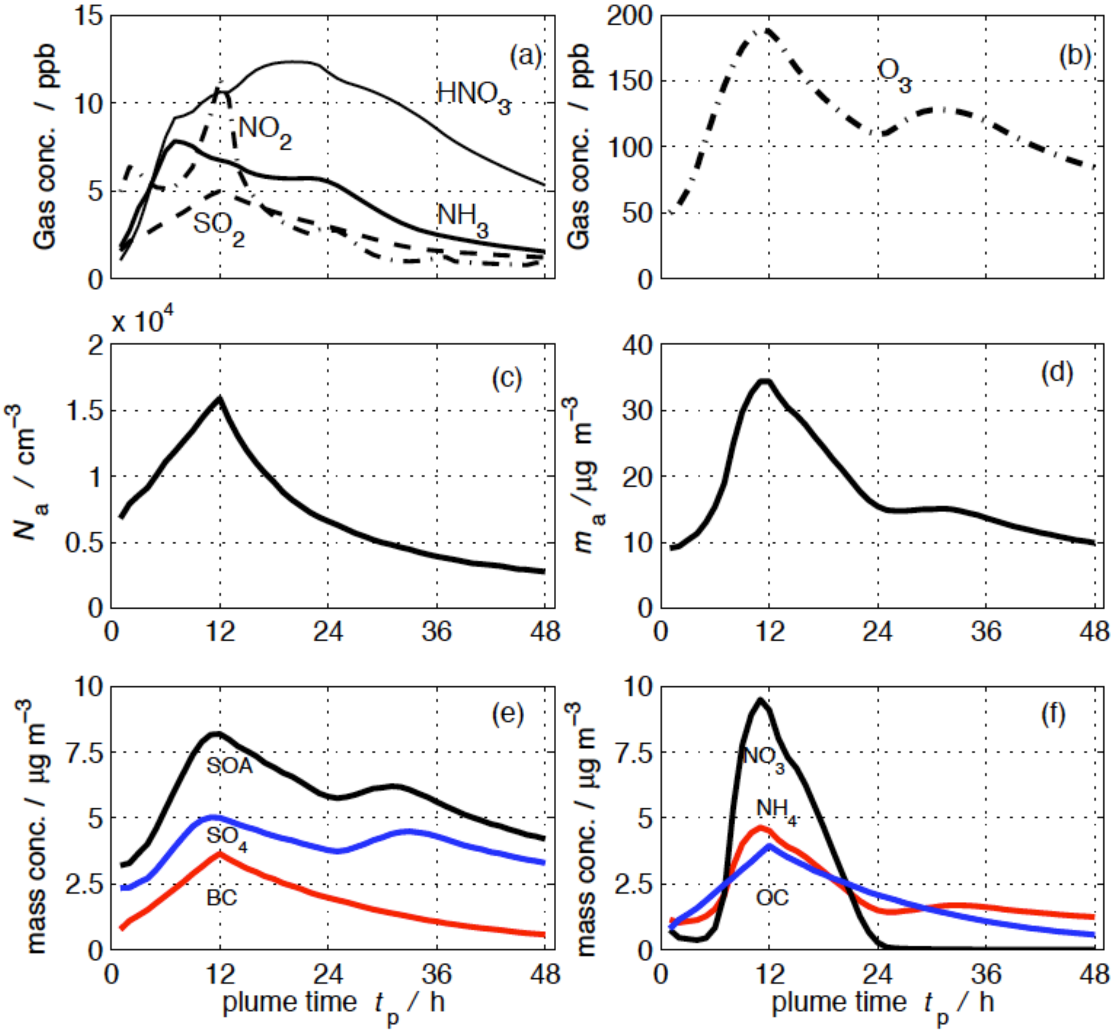

Figure S3. Urban plume environmental scenario. Time series of mixing ratios of gaseous species (panel a and b). Time series of total aerosol number concentration and aerosol mass concentration (panel c and d). Time series of mass concentrations of aerosol species (panel e and f). The aerosol species are indicated by the labels near the curves. This figure describes the urban plume scenario investigated in this work, which is the same environmental scenario studied in *Zaveri et al.,* 2010, *Ching et al.,* 2012 and *Ching et al.,* 2016. This figure is adapted from the above three publications.

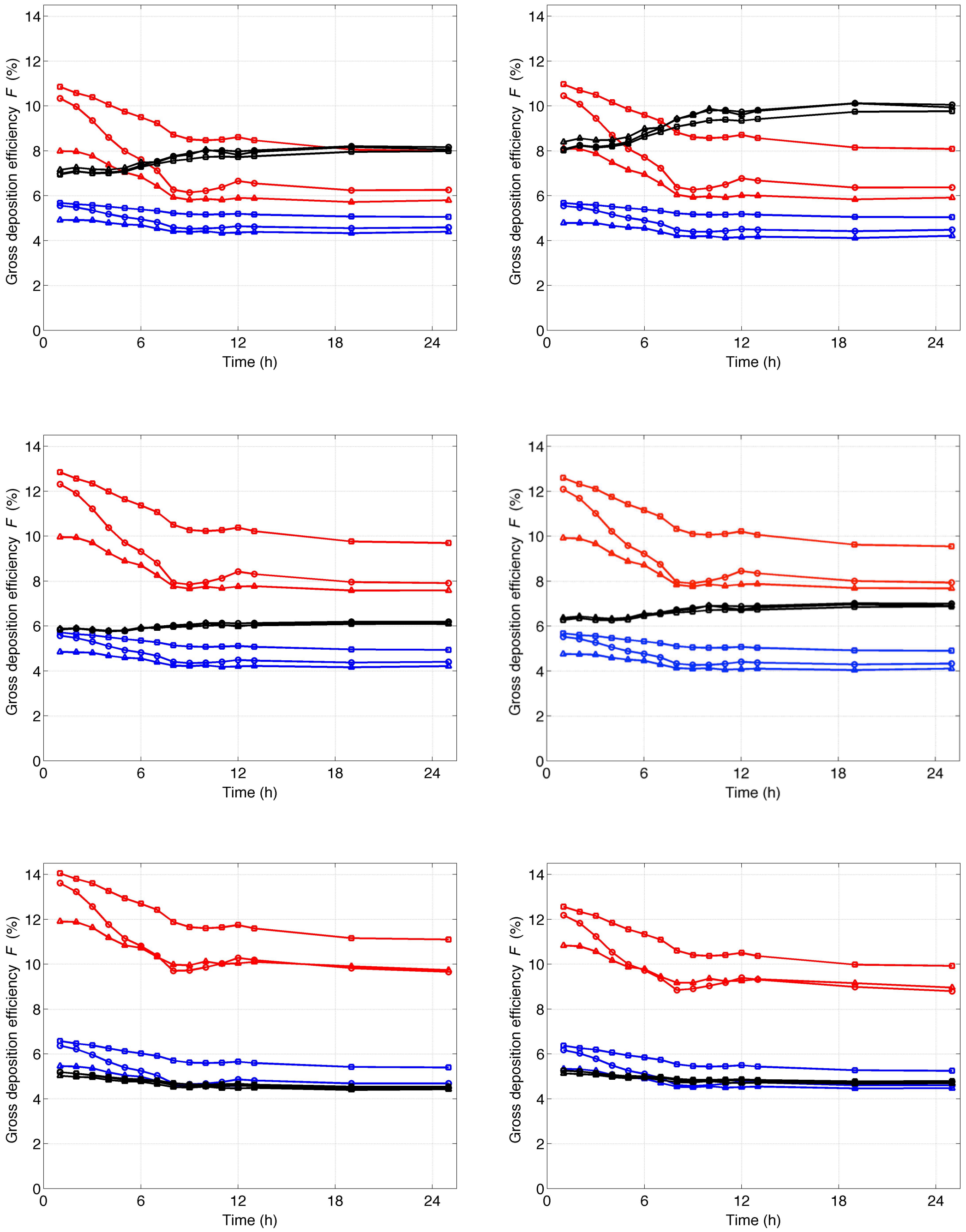

Figure S4. The gross deposition efficiency *F* of an adult male (right column) and a 10-year old child (left column) during heavy exercise (upper), light exercise (middle) and rest (bottom) with nasal inhalation verses time. Hour 0 corresponds to 6:00 in the morning. The three colors indicate the three locations in the human respiratory tract, AI (red), TB (blue) and ET (black). Three sets of simulations were performed, particle-resolved simulation (circle), composition-averaged simulation (triangle), and zero-hygroscopicity simulation (square). All simulations were performed at the relative humidity of 50%. Right middle panel is the same as Figure 3 in the main text.

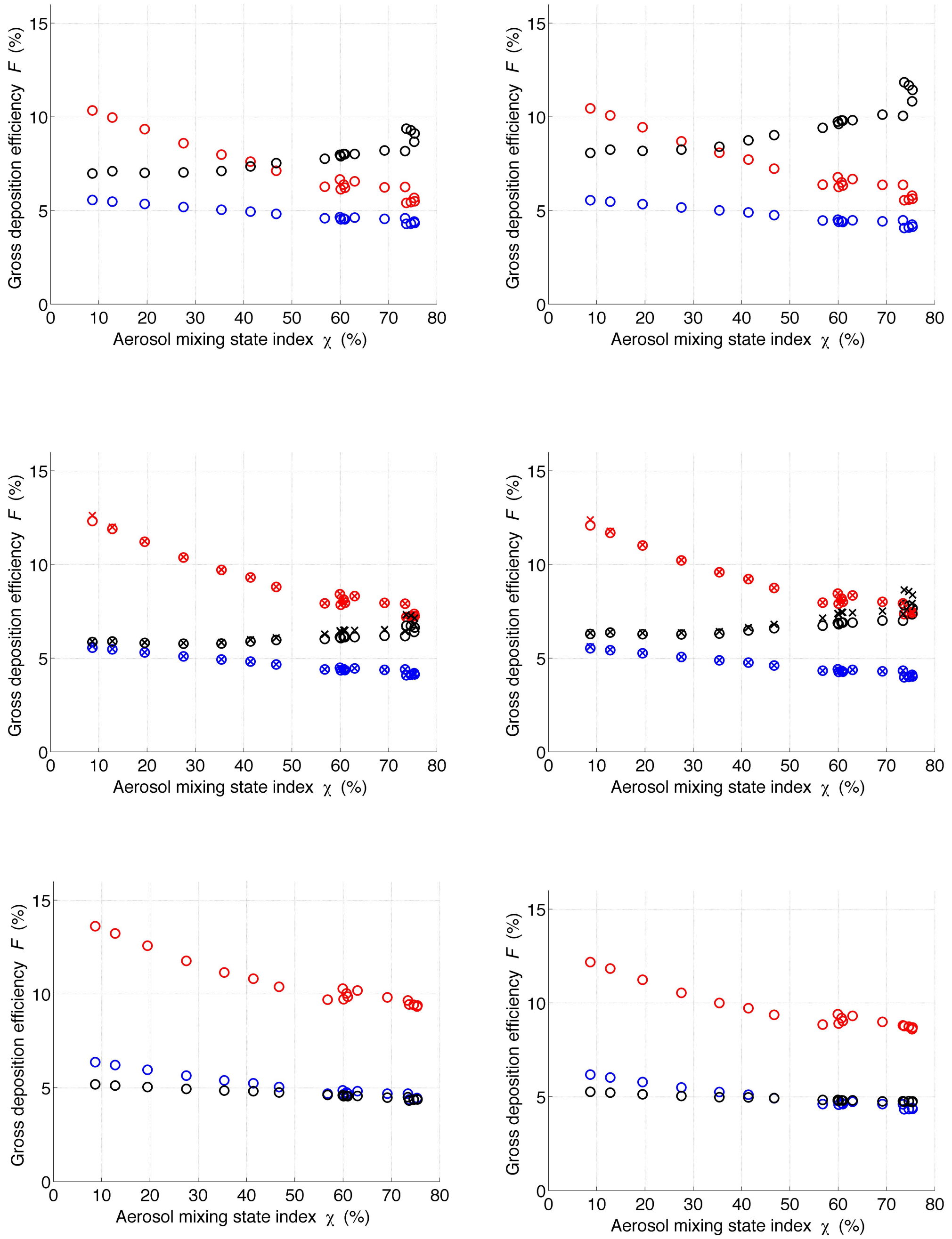

Figure S5. The gross deposition efficiency *F* of an adult male (right column) and a 10-year old child (left column) during heavy exercise (upper), light exercise (middle) and rest (bottom) with nasal inhalation verses aerosol mixing state index χ. The three colors indicate the three locations in the human respiratory tract, AI (red), TB (blue) and ET (black). All simulations were performed at the relative humidity of 50%, except that for adult male in light exercise, simulations were performed at the initial environmental relative humidities of 50% (circles) and 80% (crosses). Right middle panel is the same as Figure 4 in the main text.

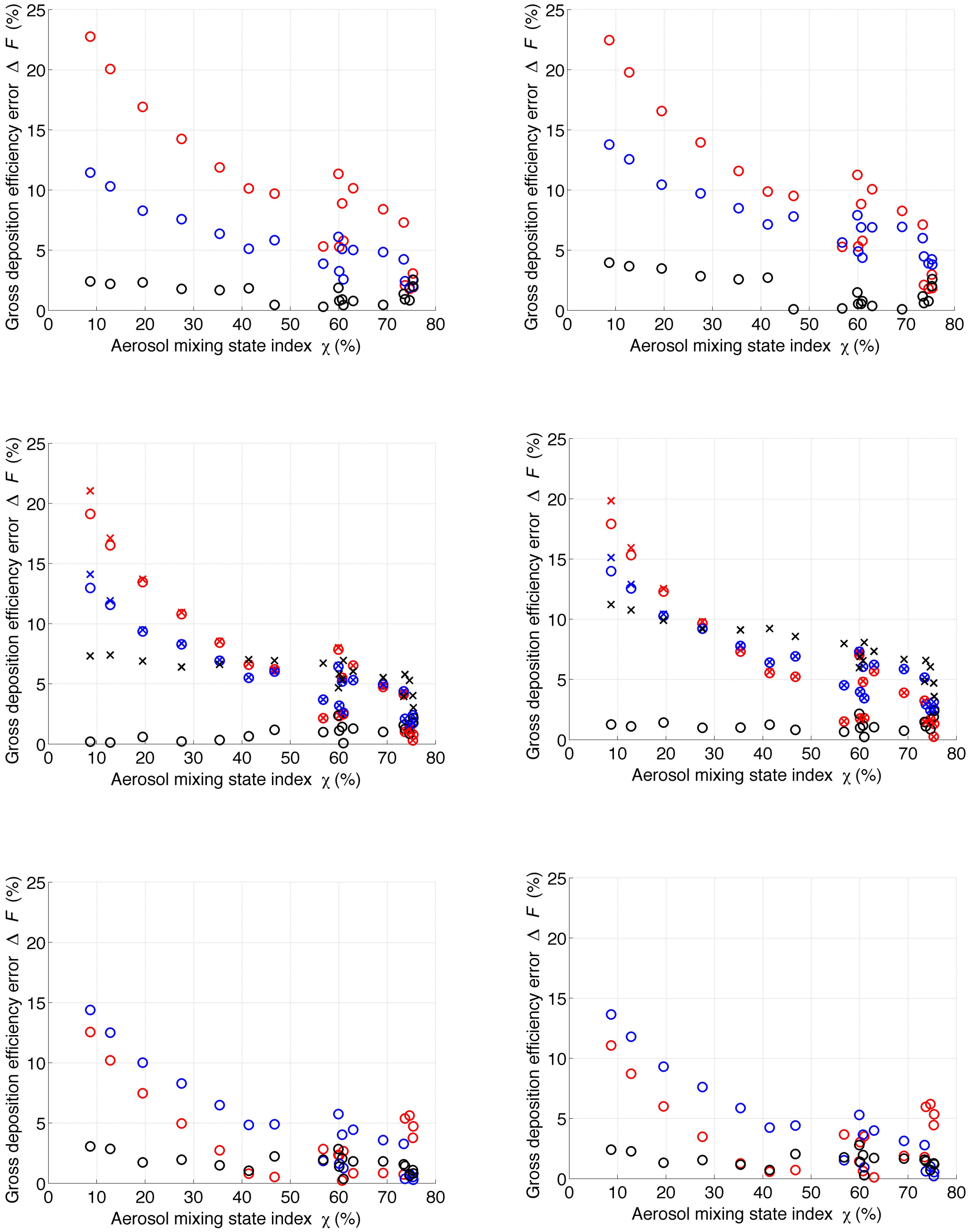

Figure S6. The error in the gross deposition efficiency ΔF of an adult male (right column) and a 10-year old child (left column) during heavy exercise (upper), light exercise (middle) and rest (bottom) with nasal inhalation verses aerosol mixing state index χ. The three colors indicate the three locations in the human respiratory tract, AI (red), TB (blue) and ET (black). All simulations were performed at the relative humidity of 50%, except that for adult male in light exercise, simulations were performed at the initial environmental relative humidities of 50% (circles) and 80% (crosses). Right middle panel is the same as Figure 5 in the main text.

Table S1. Size distribution and composition of initial conditions and background aerosols prescribed in urban plume scenario simulations. This table is adapted from *Zaveri et al.,* 2010.

Initial / Background	Number concentration (m^{-3})	Number-equivalent geometric mean diameter (μm)	Geometric standard deviation	Composition by mass
Aitken mode	1.8×10^9	0.02	1.45	49.6% $(NH_4)_2SO_4$ 49.6% SOA 0.8% BC
Accumulation mode	1.5×10^9	0.116	1.65	49.6% $(NH_4)_2SO_4$ 49.6% SOA 0.8% BC

Table S2. Size distribution and composition of aerosol emissions prescribed in urban plume scenario simulations. This table is adapted from *Zaveri et al.,* 2010.

Emission	Emission strength ($m^{-2}s^{-1}$)	Number-equivalent geometric mean diameter (μm)	Geometric standard deviation	Composition by mass
Meat cooking	9×10^6	0.0864	1.9	100% POA
Diesel vehicles	1.6×10^8	0.05	1.7	30% POA, 70% BC
Gasoline vehicles	5×10^7	0.05	1.7	80% POA, 20% BC

Table S3. Initial conditions and background mixing ratios of gaseous species prescribed in urban plume scenarios simulations. This table is adapted from *Riemer et al.,* 2009.

Chemical Species	Mixing Ratio (ppbv)
Nitrogen oxide	0.1
Nitrogen dioxide	1.0
Nitric acid	1.0
Ozone	50.0
Hydrogen peroxide	1.1
Carbon monoxide	80
Sulfur dioxide	0.8
Ammonia	0.5
Hydrogen chloride	0.7
Methane	2200
Ethane	1.0
Formaldehyde	1.2
Methanol	0.12
Methyl hydrogen peroxide	0.5
Acetaldehyde	1.0
Paraffin carbon	2.0
Acetone	1.0
Ethene	0.2
Terminal olefin carbons	2.3e-2
Internal olefin carbons	3.1e-4
Toluene	0.1
Xylene	0.1
Lumped organic nitrate	0.1
Peroxyacetyl nitrate	0.8
Higher organic acid	0.2
Higher organic peroxide	2.5e-2
Isoprene	0.5

Table S4. 12-hour average emission of gaseous species for the urban plume environmental scenarios. This table is adapted from *Riemer et al.,* 2009.

Chemical Speices	Emission (nmol m^{-2} s^{-1})
Nitrogen oxide	15.9
Nitrogen dioxide	0.84
Carbon monoxide	291.3
Sulfur dioxide	2.51
Ammonia	6.11
Formaldehyde	1.68
Methanol	0.28
Acetaldehyde	0.68
Paraffin carbon	96.0
Acetone	1.23
Ethene	7.28
Terminal olefins	2.43
Internal olefins	2.43
Toluene	4.04
Xylene	2.41
Isoprene	0.23
Alcohols	3.45

Table S5. Hygroscopicity values for model species.

Chemical species	Hygroscopicity κ
Sulfate	0.65
Nitrate	0.65
Ammonium	0.65
Black carbon	0
Primary organic aerosol (POA)	0.001
Secondary organic aerosol (SOA)	0.1

Table S6. Results of paired t-test performed for adult male in light exercise with nasal breathing. With 19 pairs of data points, we have degrees of freedom of 18. Based on two tails t-distribution table, at the significance level of 0.01, t-value = 2.88. The change in particle deposition efficiency due to (1) not considering particle hygroscopicity and; (2) considering simplified mixing state are statistically significant at 99% (except for the deposition in the ET at relative humidity of 50%, the difference in deposition efficiency due to considering simplified mixing state is not statistically significant.).

	Environmental relative humidity = 50%	*Environmental relative humidity = 80%*
AI (not considering hygroscopicity)	*t = 13.9*	*t = 12.6*
TB (not considering hygroscopicity	*t = 13.2*	*t = 12.3*
ET (not considering hygroscopicity	*t = -6.2*	*t = -6.5*
AI (considering simplified mixing state)	*t = -3.6*	*t = -3.5*
TB (considering simplified mixing state)	*t = -6.9*	*t = -6.5*
ET (considering simplified mixing state)	*t = -1.7*	*t = 21.6*

Table S7. Results of paired t-test performed for adult male and 10-year old child in light exercise with nasal breathing. With 19 pairs of data points, we have degrees of freedom of 18. Based on two tails t-distribution table, at the significance level of 0.01, t-value = 2.88. The change in particle deposition efficiency due to (1) not considering particle hygroscopicity and; (2) considering simplified mixing state are statistically significant at 99% (except for the deposition in the ET for adult male, the difference in deposition efficiency due to considering simplified mixing state is not statistically significant.).

	adult male	*10-year old child*
AI (not considering hygroscopicity)	*t = 13.9*	*t = 14.1*
TB (not considering hygroscopicity	*t = 13.2*	*t = 13.6*
ET (not considering hygroscopicity	*t = -6.2*	*t = -5.5*
AI (considering simplified mixing state)	*t = -3.6*	*t = -3.9*
TB (considering simplified mixing state)	*t = -6.9*	*t = -6.2*
ET (considering simplified mixing state)	*t = -1.7*	*t = -3.7*

Table S8. Results of paired t-test performed for adult male and 10-year old child at rest with nasal breathing. With 19 pairs of data points, we have degrees of freedom of 18. Based on two tails t-distribution table, at the significance level of 0.01, t-value = 2.88. The change in particle deposition efficiency due to (1) not considering particle hygroscopicity and; (2) considering simplified mixing state are statistically significant at 99% (except for the deposition in the Al for adult male and 10-year old child, the difference in deposition efficiency due to considering simplified mixing state is not statistically significant.).

	adult male	*10-year old child*
AI (not considering hygroscopicity)	*t = 10.0*	*t = 11.6*
TB (not considering hygroscopicity	*t = 14.2*	*t = 14.1*
ET (not considering hygroscopicity	*t = 5.2*	*t = 8.6*
AI (considering simplified mixing state)	*t = -0.15*	*t = -1.0*
TB (considering simplified mixing state)	*t = -3.9*	*t = -4.1*
ET (considering simplified mixing state)	*t = -10.6*	*t = -9.2*

Table S9. Results of paired t-test performed for adult male and 10-year old child in heavy exercise with nasal breathing. With 19 pairs of data points, we have degrees of freedom of 18. Based on two tails t-distribution table, at the significance level of 0.01, t-value = 2.88. The change in particle deposition efficiency due to (1) not considering particle hygroscopicity and; (2) considering simplified mixing state are statistically significant at 99% (except for the deposition in the ET for adult male and 10-year old child, the difference in deposition efficiency due to considering simplified mixing state is not statistically significant.).

	adult male	*10-year old child*
AI (not considering hygroscopicity)	*t = 14.4*	*t = 14.4*
TB (not considering hygroscopicity	*t = 11.8*	*t = 13.0*
ET (not considering hygroscopicity	*t = -6.9*	*t = -6.6*
AI (considering simplified mixing state)	*t = -5.1*	*t = -5.0*
TB (considering simplified mixing state)	*t = -8.6*	*t = -7.1*
ET (considering simplified mixing state)	*t = 0.8*	*t = -0.4*

Reference

Riemer, N.; West, M.; Zaveri, R. A.; Easter, R. C. Simulating the evolution of soot mixing state with a particle-resolved aerosol model. *J. Geophys. Res.: Atmos.*, **2009**, *114*(D9); DOI: 10.1029/2008JD011073

Zaveri, R. A.; Barnard, J. C.; Easter, R. C.; Riemer, N.; West, M. Particle-resolved simulation of aerosol size, composition, mixing state, and the associated optical and cloud condensation nuclei activation properties in an evolving urban plume. *J. Geophys. Res. Atmos.*, **2010**, 115(D17).

Ching, J.; Riemer, N.; West, M. Impacts of black carbon mixing state on black carbon nucleation scavenging: Insights from a particle-resolved model. *J. Geophys. Res.: Atmos.*, **2012**, 117(D23).

Ching, J.; Riemer, N.; West, M. Black carbon mixing state impacts on cloud microphysical properties: Effects of aerosol plume and environmental conditions. *J. Geophys. Res.: Atmos.*, **2016**, 121(10), 5990-6013.
